# Effects of *Pereskia aculeate* Miller Petroleum Ether Extract on Complete Freund’s Adjuvant-Induced Rheumatoid Arthritis in Rats and its Potential Molecular Mechanisms

**DOI:** 10.3389/fphar.2022.869810

**Published:** 2022-05-09

**Authors:** Yifei Chen, Kaifei Liu, Yingyuan Qin, Suyi Chen, Guokai Guan, Yao Huang, Yu Chen, Zhixian Mo

**Affiliations:** ^1^ School of Traditional Chinese Medicine, Southern Medical University, Guangzhou, China; ^2^ School of Pharmacy, Guilin Medical University, Guilin, China; ^3^ Nephrology, Guilin TCM Hospital of China, Guilin, China

**Keywords:** petroleum ether extract of *P. aculeate* Miller, rheumatoid arthritis, p38/MAPK signaling pathway, inflammation, ethnomedicine

## Abstract

**Objective:** To investigate the therapeutic effect of petroleum ether extract of *P. aculeate* Miller (PEEP) on rheumatoid arthritis (RA).

**Methods:** *In vitro*: The Cell Counting Kit-8 (CCK-8) was used to detect cell activity and select the optimal concentration of the extract; the effective site was screened by nitric oxide (NO) colorimetric method and Q-PCR method; the expression of p38, p-p38, p-MK2, and Tristetraprolin (TTP) in RAW 264.7 cells were detected by Western blot. *In vivo*: The rat model was established by complete Freund’s adjuvant (CFA). The different doses of PEEP on CFA rats were observed with life status, paw swelling, spleen index, X-ray, Hematoxylin eosin (HE) staining; the secretion of Tumor necrosis factor α (TNF-α), interleukin-6 (IL-6) and Prostaglandin E2 (PGE_2_) were detected by Enzyme linked immunosorbent assay (ELISA); the expressions of p38, p-p38, p-MK2, and TTP in the ankle joints of CFA rats were detected by Western blot.

**Result:** *In vitro*: PEEP, Ethyl Acetate Extract of *P.* acul*eate* Miller (EEEP), N-butanol Extract of *P. aculeate* Miller (BEEP) have no toxic effects on RAW264.7 macrophages. PEEP, EEEP, and BEEP reduce the secretion of NO in RAW264.7 cells induced by lipopolysaccharide (LPS), only PEEP significantly inhibited the mRNA expression levels of inflammatory factors TNF-α and IL-6; PEEP-dependently reduce the secretion of TNF-α and IL-6, decrease the expression of p-p38 and p-MK2, and the level of TTP phosphorylation in LPS-induced RAW264.7 cells. *In vivo*: PEEP improve the living conditions of CFA rats, reduce foot swelling, spleen index, bone surface erosion and joint space narrowing; reduce the formation of synovial cells, inflammatory cells and pannus in the foot and ankle joints. PEEP reduce the secretion of TNF-α, IL-6, PGE_2_ in rat serum, downregulate the expression of p-p38 and p-MK2 in the ankle joint, and reduce the phosphorylation of TTP.

**Conclusion:** PEEP improve the living conditions of CFA rats, reduce the degree of foot swelling, protect immune organs, reduce inflammatory cell infiltration, cartilage damage, pannus formation, reduce inflammation and RA damage. The mechanism through regulating the signal pathway of p38 mitogen-activated protein kinase (p38/MAPK), which reduces the release of TNF-α, IL-6, and PGE_2_ in the serum.

## 1 Introduction

Rheumatoid arthritis is an autoimmune disease characterized by polyarthritis, progressive joint damage, and swelling deformities (Pu et al., 2016; Sparks, 2019; Shi et al., 2020). The pathological features of RA are joint synovial lesions, including excessive synovial cell proliferation, inflammatory cell infiltration, pannus formation, joint bone and cartilage destruction (Aletaha et al., 2011; Xing et al., 2018). According to relevant investigations and studies, the incidence of this disease worldwide is as high as 1%–2%. There are more than 5 million RA patients in China, most of whom are in the 30–50 years old age group. It is the second largest cause of disability in the Chinese population (van der Woude and van der Helm-van Mil, 2018). It can quickly develop into multi-system inflammation and irreversible joint damage, leading to a decline in quality of life, disability and death without proper treatment (Malmstrom et al., 2017). Because the etiology and pathogenesis of RA are not clearly understood (Mcinnes and Schett, 2011). The targets and methods of clinical treatment are not clear, which brings certain difficulties to treatment. There are no highly targeted therapeutic drugs in clinical practice, the treatment of RA still from the aspects of analgesia, anti-inflammatory, preventing or reduce joint deformation and damage and increasing joint mobility (Yuan et al., 2015; Shams et al., 2021). These drugs reduce joint inflammation, inhibit the development of lesions and irreversible bone destruction, but long-term use of these drugs lead to immune system decline, bone marrow suppression, liver and kidney function damage, gastrointestinal function decline, cartilage degeneration, infection (Abbasi et al., 2019; Koller et al., 2019). Therefore, it is clinically necessary to find effective drugs with low toxicity to treat RA. In recent years, ethnomedicine and some natural medicine products have attracted more and more researchers’ attention because of their unique safe and effective pharmacological activities (Jang et al., 2014). Ethnomedicine can be a new resource for the treatment of rheumatoid arthritis with more and more evidences.

*P. aculeata* Mill is a plant of the genus Pereskia in the Cactaceae family, also known as leaf cactus and tiger thorn. It is mainly produced in Yunnan, Guangdong, Guangxi and other regions south of the Yangtze River. It has a long history of folk medicine, with low side effects, and anti-inflammatory, antioxidant, antibacterial and other pharmacological activities (Tan et al., 2005; Kazama et al., 2012; Pinto and Scio, 2014; Pinto et al., 2015). Its medicinal liquor can treat bruises and rib pain. According to reports in the literature, there are as many as 30 chemical components in the essential oil of *P. aculeata* Mill leaves, among which the higher content includes phytol, palmitic acid, linoleic acid (Souza et al., 2014). In addition, the plant essential oil has the highest content of oxidized sesquiterpenes (44.92%), acorus spirenone comes next. The current research on the pharmacological effects of *P. aculeata* Mill mainly focuses on analgesia, cardiovascular protection and other related aspects. There are few studies on its anti-RA effect (Peng et al., 2018). The previous research show that *P. aculeata* Mill ethanol extract inhibited acetic acid-induced increase in vascular permeability and writhing behavior of mice in acetic acid-induced writhing reaction experiment, reduce the swelling of the feet of CFA rats and the level of IL-6 and other inflammatory cytokines in the rat serum, inhibit LPS-induced inflammatory response in RAW264.7 macrophages (Kanno et al., 2006).

This study aimed to screen effective sites by establishing an LPS-induced RAW264.7 macrophage inflammation model, and through the FCA rat model to evaluate the therapeutic effect and possible mechanism of PEEP, provide a more adequate theoretical basis for the development and utilization of PEEP.

## 2 Materials and Methods

### 2.1 Cell Culture and Laboratory Animals

Mouse macrophages RAW264.7 were from the Pharmacology Laboratory of Guilin Medical University, China, and were preserved by our laboratory for future generations.

Healthy female SD rats, SPF grade, 60 rats, weight (200 ± 20 g), provided by Hunan SJA Laboratory Animal Co., Ltd., license SCXK Xiang 2016-0002. Rats were kept at a temperature of 21°C–25°C and a relative humidity of 50%–60%. Rats in each group eat and drink freely.

### 2.2 Main Experimental Reagents

#### 2.2.1 Main Drugs and Reagents

*P. aculeata* Mill petroleum ether extract; NO kit (Wuhan, Elabscience Biotechnology Co., Ltd.); p38MAPK antibody, p-p38MAPK antibody, p-MK2 antibody and TTP antibody (CST, Miami, FL, United States); β-Actin (Proteintech company, Chicago, IL, United States); Primary antibody and secondary antibody diluent (China, Beyotime Biotechnology); Rainbow marker (Thermo Fisher Scientific, Waltham, MA, United States); Fetal Bovine Serum (GIBCO Company, Waltham, MA, United States); Cell protein lysate, Phosphatase inhibitor (Beijing, CWBIO Biotechnology Co., Ltd.); Lipopolysaccharide (Sigma-Aldrich Reagent Company, United States); CCK-8 kit (Japan, DOJIDO company); AceQ qPCR Green Master Mix, HiScript QRT SuperMIX (Nanjing, Vazyme Biotech Co., Ltd.); 1M Tirs-Hcl 6.8, 1M Tirs-Hcl 8.8, PBS (Phosphate buffer asline), Glycine, SDS, Tris (China, Solarbio Biotechnology Co., Ltd.); Primer (Shenzhen, BGI Technology Co., Ltd.). PEEP (Laboratory Preparation); Freund’s complete adjuvant (SIGMA-ALDRICH, St. Louis, MI, United States); Dexamethasone (DEX) (Guangdong, Sancai Shiqi Pharmaceutical Co., Ltd.); Rat PGE_2_, IL-6 and TNF-α kits (Wuhan, Elabscience Biotechnology Co., Ltd.); 4% tissue fixative (China, Solarbio Life Sciences Co., Ltd.) (Tables 1, 2).

**TABLE 1:** Primer names and sequences.

Primer name	Primer sequence
GAPDH-F	5′-AGA​AGG​CTG​GGG​CTC​ATT​TG-3′
GAPDH-R	5′-AGG​GGC​CAT​CCA​CAG​TCT​TC-3′
IL-6-F	5′-CCG​GAG​AGG​AGA​CTT​CAC​AG-3′
IL-6-R	5′-TCC​ACG​ATT​TCC​CAG​AGA​AC-3′
TNF-α-F	5′-CGT​CAG​CCG​ATT​TGC​TAT​CT-3′
TNF-α-R	5′-CGG​ACT​CCG​CAA​AGT​CTA​AG-3′

**TABLE 2:** Q-PCR reaction system.

Reaction system	Volume (μl)
SYBR	5
Forward primer	1
Reverse primer	1
cDNA	1
ddH2O	2

### 2.3 Main Experimental Equipment

Epoch microplate reader (Biotek, Winooski, VT, United States); Small desktop refrigerated centrifuge, Liquid nitrogen storage tank (Thermo Fisher Scientific, Waltham, MA, United States); Protein electrophoresis, Electric transfer system, Gel imaging analysis system (Bio-Rad, Hercules, CA, United States); Constant temperature water bath (Shanghai, Baoling Instrument Equipment Co., Ltd.); Carbon dioxide constant temperature incubator, Ice machine (Sanyo, Japan); Ultra-clean workbench, High Pressure Steam Sterilizer (Shandong, BIOBASE); Fluorescence quantitative PCR machine (IT-IS company, United Kingdom); Foot swelling degree measuring instrument (Ugo Basile company, Italy); Small animal live optical imaging system (Shanghai, PerkinElmer Enterprise Management Co., Ltd.); Pipette gun (Eppendorf, Germany); Electric heating constant temperature sink (Shanghai, Huitai Instrument Manufacturing Co., Ltd.).

### 2.4 Experimental Method

#### 2.4.1 Preparation of Effective Parts of *P. aculeata* Mill

We took 1,000 ml of *P. aculeata* Mill ethanol extract, filtered with a qualitative filter paper circle, and then concentrated it with a rotary evaporator, added water to suspend the extract. Extract with petroleum ether, ethyl acetate and n-butanol, recover the solvent and evaporate to dryness to obtain PEEP 2.415 g, EEEP 3.598 g and BEEP 3.473 g.

#### 2.4.2 Screening of Effective Parts of *P. aculeata* Mill

RAW264.7 macrophages were used to screen the effective site of *P. aculeata* Mill. The concentration of each extraction part of *P. aculeata* Mill is setting as follows: PEEP: 0, 15, 30, 60, 120, and 240 μg/ml; EEEP: 0, 5, 15, 30, and 60 μg/ml, BEEP: 0, 10, 20, 40, and 80 μg/ml. The effects of PEEP, EEEP, and BEEP on the viability of RAW264.7 macrophages were detected by CCK-8, calculate the survival rate of each group of cells according to the formula.

The experiment set up normal group, LPS group (1 μg/ml), LPS + DEX group (1 × 10^−4^ M), LPS + PEEP (60 μg/ml), LPS + EEEP (30 μg/ml), LPS + BEEP (40 μg/ml). The effect of each extract part on NO content in RAW264.7 macrophages stimulated by LPS was detected according to the instructions in Wuhan Elabscience Biotechnology Co., Ltd. NO kit, and Q-PCR was used to detect the levels of TNF-α and IL-6 in RAW264.7 macrophages stimulated by LPS in each extraction site to screen the most effective site.

(1) The configuration of the Q-PCR reaction system:
(2) The relative expression formula of the target gene is:

$${\left[2^{Ct\left(\text{target}\;\text{gene}\right)}/2^{Ct\left(\text{reference}\;\text{gene}\right)}\right]}^{-1}=2^{-\Delta Ct}$$

According to the previous experimental results, the effect of PEEP (15, 30, and 60 μg/ml) on NO content in RAW264.7 macrophages stimulated by LPS was detected. Q-PCR was used to detect the effect of PEEP (15, 30 and 60 μg/ml) on the expression levels of TNF-α and IL-6 in LPS-induced RAW264.7 macrophages. And the effect of PEEP (15, 30 and 60 μg/ml) on p38/MAPK signaling pathway in LPS-induced RAW264.7 macrophages was detected by western blot.

#### 2.4.3 Preparation and Grouping of CFA Rat Model

After adaptive feeding for 7 days, 60 rats were randomly divided into six groups: blank group, model group, L-PEEP (16 mg/kg) group, M-PEEP (32 mg/kg) group, H-PEEP (64 mg/kg) group, DEX (0.5 mg/kg) group. Except for the 6 rats in the blank group, the rats in the other groups all used Freund’s complete adjuvant to prepare CFA models. After routine disinfection, straighten the right paw of the rat, and use a 1 ml sterile syringe to inject 0.1 ml of Freund’s complete adjuvant into the center of the plantar of each rat.

#### 2.4.4 Method of Administration

According to the characteristics of the CFA model (Chang et al., 2011), the foot swelling at the model site reached its peak (acute inflammatory response) at 18–27 h, and it continued for 10 days and then gradually reduced; secondary lesions generally appeared at 11 days after inflammation. Combined with pre-experimental results, in this experiment, the drug was started 14 days after the model was established, and the drug was administered continuously for 10 days.

#### 2.4.5 Measurement of Rat Foot Swelling Degree

Observe each group at 0, 9, 18, and 27 h, d 3, d 5, d 9, d 11, d 13, d 15, d 18, d 20, d 22, and d 24 after inflammation. The swelling of the hind paw of the rat was measured, and the swelling degree of the right hind paw and the swelling degree of the left hind paw on the non-inflammatory side were measured with a foot swelling meter.

#### 2.4.6 X-Rays to Observe Changes in Cartilage and Bone Structure

After 10 days of administration, the rats in each group were observed to undergo changes in cartilage and bone structure by X-ray. The rats in each group were intraperitoneally injected with 10% chloral hydrate. After anesthesia, take the supine position and transfer to the small animal *in vivo* imaging device, and take pictures of the left and right feet of each group of rats in turn.

#### 2.4.7 Determination of Spleen Index

After the rats were sacrificed, the abdominal cavity of the rats was cut open quickly and the spleens were cut out. The spleens were quickly rinsed with cold PBS, and the remaining liquid was soaked up with filter paper and weighed to calculate the spleen index of the rats.

Spleen index=spleen weight/corresponding rat's weight

#### 2.4.8 Determination of Serum TNF-α, IL-6, and PGE_2_ Levels in Rats

After the last administration, the rats were fasted for 24 h. After weighing, the rats in each group were blood collected from the vein behind the eyeball and centrifuged to extract serum. They were allowed to stand in a refrigerator at 4°C for 2 h, centrifuged at 4,000 rpm for 10 min, and the supernatant was collected and used for experiment. The concentration of TNF-α, IL-6, and PGE_2_ in serum was detected by ELISA method, and the operation was performed according to the kit instructions.

#### 2.4.9 HE Staining to Observe the Pathological Changes of the Foot and Ankle Joints

After removing the soft tissue around the right hind ankle of the rat, it was immersed in 4% tissue fixative for 24 h, and sent to the Department of Pathology, Affiliated Hospital of Guilin Medical College to make HE stained sections. The slices were taken to capture images under a 200×, 400× field of view.

#### 2.4.10 Western Blot Detection

Total protein extracted from cells and ankle joints, and then separated by SDS-PAGE gel electrophoresis to bromophenol blue to the bottom of the separation gel. The protein was transferred to PVDF membrane and blocked with 5% skimmed milk powder for 1.5 h. Incubate overnight with primary antibodies β-actin, p38, p-p38, p-MK2, and TTP at 4°C. Incubate for 1.5 h with goat anti-mouse IgG and goat anti-rabbit IgG. All experiments were repeated 3 times.

### 2.5 Statistical Analysis

SPSS 20.0 was used for data analysis, GraphPad Prism 6.0 was used for graphing, and the data were expressed as mean ± standard deviation (mean ± S.E.M). The comparison between groups was performed by one-way analysis of variance. *p* < 0.05 and *p* < 0.01 were considered statistically different.

## 3 Results

### 3.1 CCK-8 Detects the Effect of Each Extraction Part of *P. aculeata* Mill on the Viability of RAW264.7 Macrophages

**FIGURE 1:**
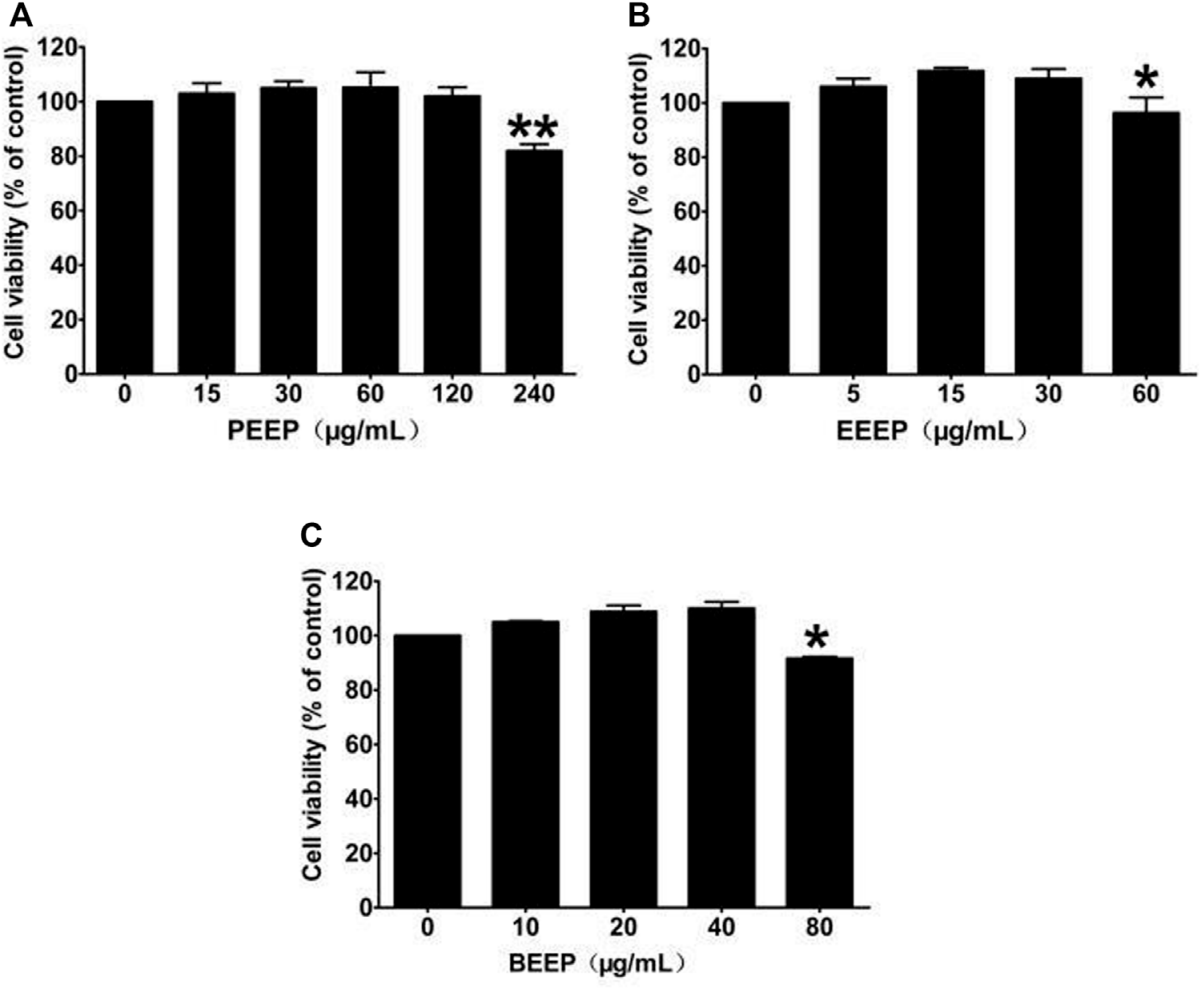
Effects of different concentrations of PEEP, EEEP, and BEEP on cell viability in RAW264.7 cells. Results were exhibited as the means ± S.E.M. of three independent experiments. ^*^ *p* < 0.05, ^**^ *p* < 0.01 vs. Normal group.

The CCK-8 experiment was used to detect the effects of PEEP, EEEP, and BEEP on the survival rate of RAW264.7 macrophages. PEEP (0, 15, 30, and 60 μg/ml), EEEP (0, 5, 15, and 30 μg/ml) and BEEP (0, 10, 20, and 40 μg/ml) concentration survival rate is similar to the blank control group (Figures 1A–C). The results illustrated that each extraction site has no toxic and side effects on RAW264.7 macrophages within the designed concentration. Therefore, PEEP (60 μg/ml), EEEP (30 μg/ml), and BEEP (40 μg/ml) were selected as the highest concentrations for subsequent experiments.

### 3.2 The Effect of the Extracts of *P. aculeata* Mill on the Expression of Nitric Oxide in RAW264.7 Macrophages Induced by Lipopolysaccharide

**FIGURE 2:**
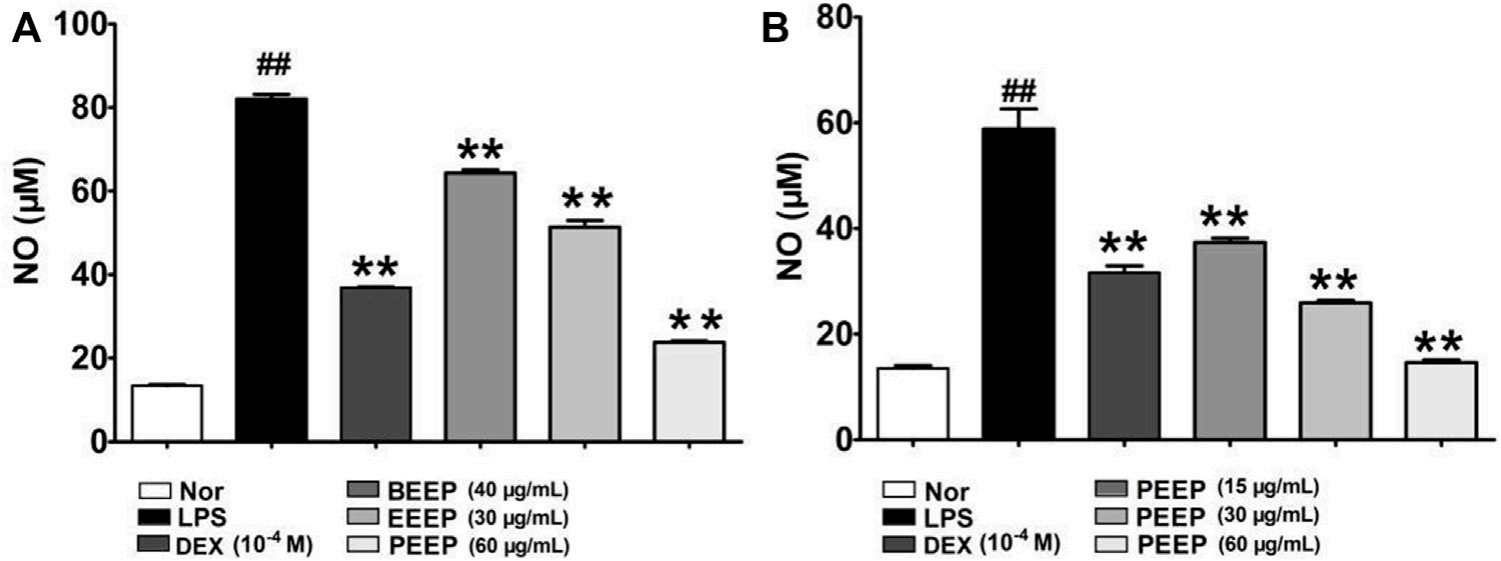
Effects of different concentrations of PEEP, EEEP and BEEP on cell viability in RAW264.7 macrophages. **(A)** Effect of PEEP (60 μg/ml), EEEP (30 μg/ml) and BEEP (40 μg/ml) on the content of NO induced by LPS in RAW264.7 macrophages. **(B)** Effect of PEEP (15, 30 and 60 μg/ml) on the content of NO induced by LPS in RAW264.7 macrophages. Results were exhibited as the means ± S.E.M. of three independent experiments. ^##^ *p* < 0.01 vs. Normal; ^*^ *p* < 0.05, ^**^ *p* <0.01 vs. LPS group.

LPS stimulate macrophages to express nitric oxide synthase (iNOS) and release NO (Chen et al., 2014). Therefore, the effect of each extraction site of *P. aculeata* Mill on LPS-induced NO expression in RAW264.7 macrophages was investigated by NO colorimetry. The expression level of No in RAW264.7 macrophages induced by LPS was significantly reduced (*p* < 0.01) through the intervention of each extraction site of *P. aculeata* Mill (Figure 2A). The inhibitory effect of PEEP on NO is stronger than other parts, and it has the most significant anti-inflammatory potential. PEEP (15, 30 and 60 μg/ml) significantly reduce the expression of NO in RAW264.7 macrophages induced by LPS when compared with the model group, and in concentration-dependent (Figure 2B).

### 3.3 Effects of the Extracts of *P. aculeata* Mill on the Expression of TNF-α and IL-6 in RAW264.7 Macrophages Induced by Lipopolysaccharide

**FIGURE 3:**
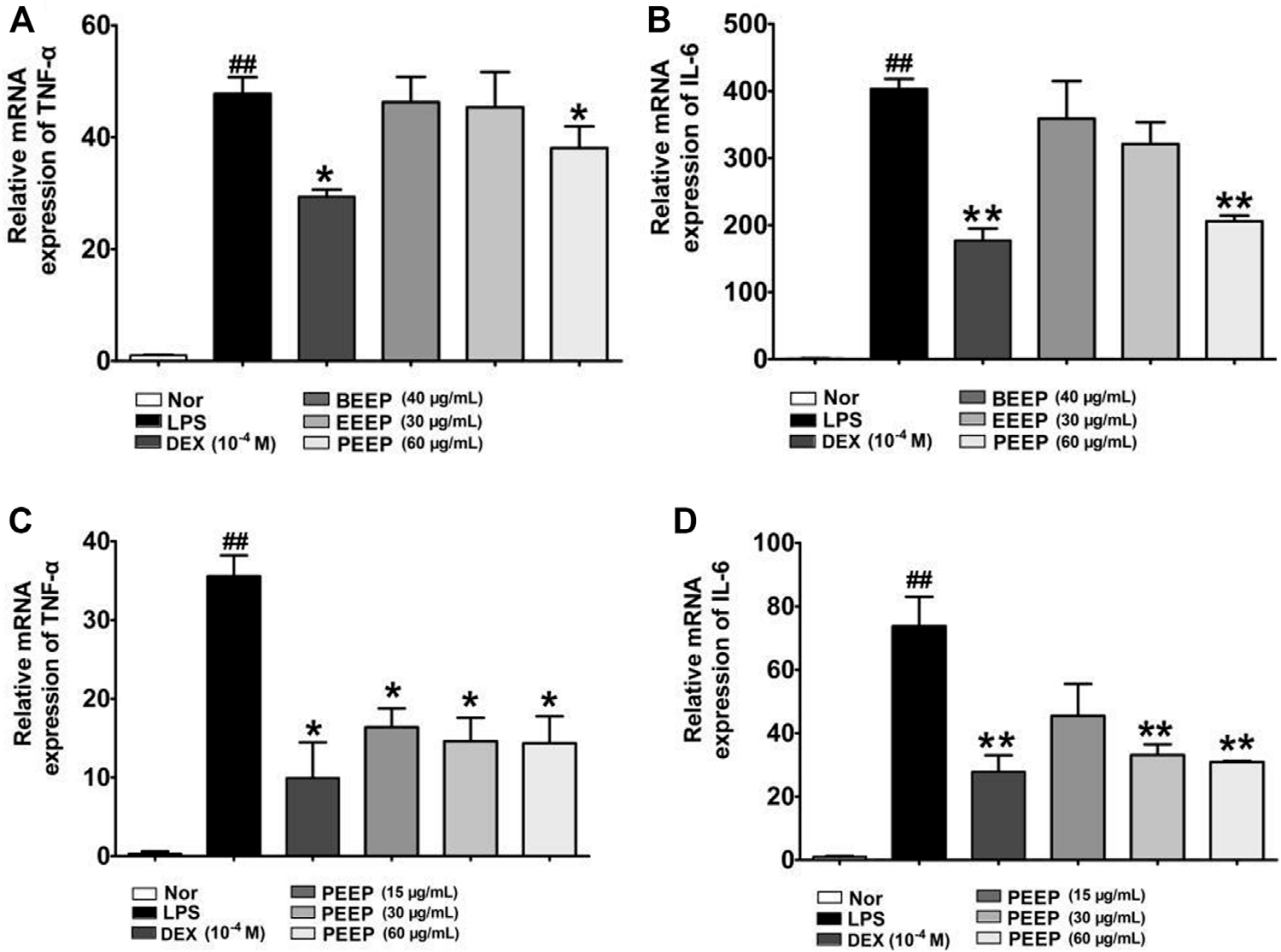
Effect of PEEP, EEEP and BEEP on the expressions of inflammatory factors in RAW264.7 macrophages induced by LPS. **(A)** The effect of PEEP (60 μg/ml), EEEP (30 μg/ml) or BEEP (40 μg/ml) on mRNA expressions of TNF-α were detected using Q-PCR assays. **(B)** The effect of PEEP (60 μg/ml), EEEP (30 μg/ml) or BEEP (40 μg/ml) on mRNA expressions of IL-6 were detected using Q-PCR assays. **(C)** The effect of PEEP (15, 30 and 60 μg/ml) on mRNA expressions of TNF-α were detected using Q-PCR assays. **(D)** The effect of PEEP (15, 30 and 60 μg/ml) on mRNA expressions of IL-6 were detected using Q-PCR assays. The data were expressed as the means ± S.E.M. of three independent experiments. ^##^ *p* < 0.01 vs. Normal group; ^*^ *p* < 0.05, ^**^ *p* < 0.01 vs. LPS group.

Macrophages play an important role in the pathogenesis of RA by secreting TNF-α, IL-6 and other inflammatory cytokines (Kanno et al., 2006). The related expression results are shown in Figures 3A,B. PEEP reduce the mRNA expression levels of TNF-α and IL-6 in RAW264.7 macrophages induced by LPS when compared with the model group (*p* < 0.05, *p* < 0.01). However, the inhibition of TNF-α and IL-6 by EEEP and BEEP was not statistically significant (*p* > 0.05). PEEP (15, 30 and 60 μg/ml) decreased the mRNA levels of TNF-α and IL-6 in LPS-induced RAW264.7 macrophages (*p* < 0.05, *p* < 0.01) in a concentration-dependent manner (Figures 3C,D). The above results indicate that PEEP significantly reduce the level of TNF-α and IL-6 mRNA in activated RAW264.7 macrophages, and the concentration increases, the lower the mRNA level. Therefore, based on the experimental results, PEEP (15, 30 and 60 μg/ml) was selected for subsequent experiments.

### 3.4 Effect of PEEP on the p38/MAPK Signaling Pathway of Lipopolysaccharide-Induced RAW264.7 Macrophages

**FIGURE 4:**
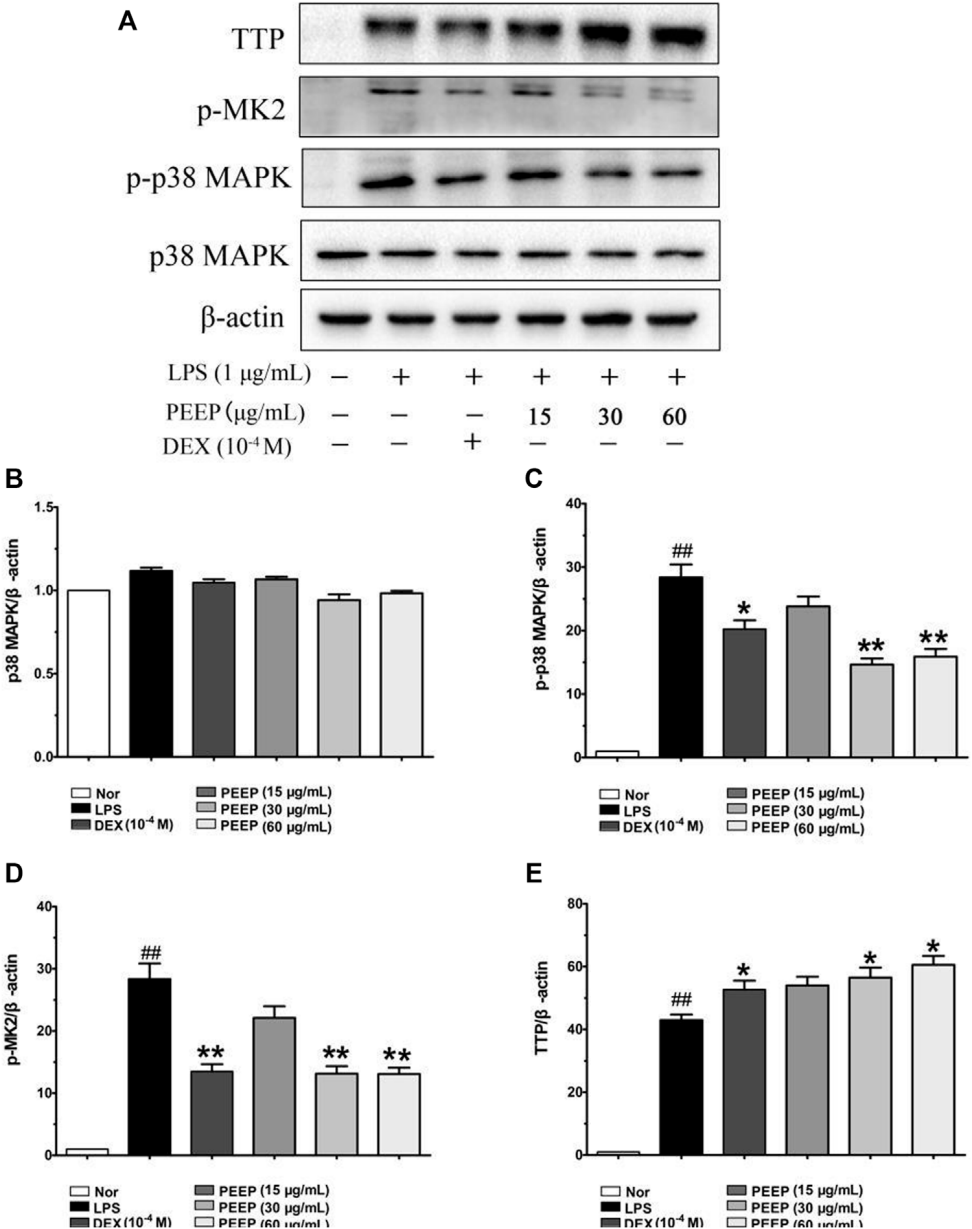
Effect of PEEP on p38, p-p38, p-MK2, and TTP expressions induced by LPS in RAW264.7 macrophages. **(A)** The protein expressions of p38, p-p38, p-MK2, and TTP were detected by using western blot analysis. **(B)** Quantitative analysis of gray value of p38 was performed in several groups with β-actin as loading control. **(C)** Quantitative analysis of gray value of p-p38 was performed in several groups with β-actin as loading control. **(D)** Quantitative analysis of gray value of p-MK2 was performed in several groups with β-actin as loading control. **(E)** Quantitative analysis of gray value of TTP was performed in several groups 27 with β-actin as loading control. Data represent the mean ± S.E.M. of three independent experiments. ^*##*^ *p* < 0.01 vs. Normal group; ^*^ *p* < 0.05, ^**^ *p* < 0.01. vs. LPS group.

The p38/MAPK pathway is closely related to the regulation of inflammatory response. It is an important mediator involved in the expression of a variety of genes in the stress process. After activation, it moves from the cytoplasm to the nucleus, and interacts with the corresponding transcription factors to control the transcription of a variety of genes (Roussel et al., 2010), it plays a key role in the signal transduction of a variety of cytokine synthesis induced by LPS. Therefore, we analyzed the expression of p38, p-p38, p-MK2, and TTP protein in order to further explore the molecular mechanism of PEEP anti-inflammatory. PEEP downregulated the expression levels of p-p38, p-MK2 and increased the expression level of TTP in LPS-induced RAW264.7 macrophages when compared with the model group (*p* < 0.01, *p* < 0.05) in a concentration-dependent (Figure 4).

### 3.5 General Observation of Rats in Each Group

The rats with normal appetite and active behavior before CFA injection. The skin of the feet of CFA rats began to become red, and the modeled feet were swollen after inflammation for 2 days. And the primary paws of CFA rats were significantly more swollen, and movement was significantly restricted, the secondary paws were not significantly swollen, and appetite decreased slightly after inflammation for 7 days when compared with the control group. Rats in the PEEP medium and high dose group had better eating conditions, and relieved the swelling of the feet after 10 days of administration when compared with the model group. No abnormally dead rats were found.

### 3.6 The Effect of PEEP on the Swelling Degree of Complete Freund’s Adjuvant Rats’ Feet

**FIGURE 5:**
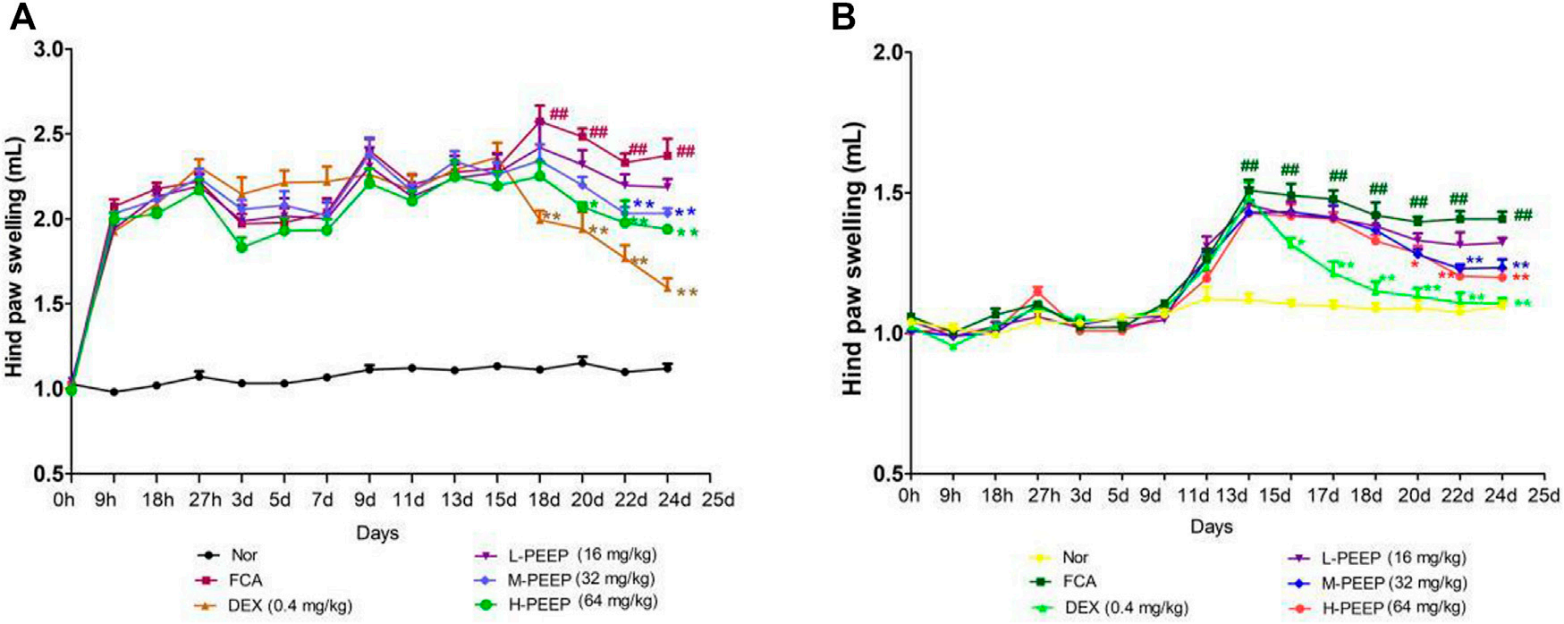
Effects of different doses of PEEP on swelling of paw in CFA rats. **(A)**Effects of different doses of PEEP on swelling of right hind paw in CFA rats. **(B)** Effects of different doses of PEEP on swelling of left hind paw in CFA rats. ^##^ *p* < 0.01 vs. Normal group; ^*^ *p* < 0.05, ^**^ *p* < 0.01 vs. CFA group.

Combined with the characteristics of the CFA model (Chang et al., 2011). The swelling degree of the right hind foot of the model group CFA rats gradually reached a peak after 15 days of modeling when compared with the blank group (*p* < 0.01). The swelling of the right hind foot of rats in the M-PEEP (32 mg/kg) group and H-PEEP (64 mg/kg) group decreased significantly after 10 days of administration when compared with the model group (*p* < 0.01). The CFA rats in the model group developed secondary swelling of the left hind foot 11 days after modeling when compared with the blank group (*p* < 0.01). The swelling of the left hind foot of the rats in the M-PEEP (32 mg/kg) group and H-PEEP (64 mg/kg) group decreased significantly after 10 days of administration when compared with the model group (*p* < 0.01). The results show that the petroleum ether site significantly reduce the degree of foot swelling in CFA model rats (Figures 5A,B).

### 3.7 The Effect of PEEP on the Spleen Index of Complete Freund’s Adjuvant Rats

The results are presented in Table 3, the spleen index of the model group increased when compared with the blank group (*p <* 0.05). The spleen index of M-PEEP (32 mg/kg) group and H-PEEP (64 mg/kg) group was significantly lower than model group (*p <* 0.05). The spleen index of the L-PEEP (16 mg/kg) group was lower than model group, but there is no significant difference (*p* > 0.05).

**TABLE 3:** The spleen index of CFA rats.

Group	Dose (mg/kg)	Spleen index (mg/kg)
Nor	0	2.52 ± 0.11
CFA	0	2.99 ± 0.10^#^
DEX	0.5	2.68 ± 0.07^*^
L-PEEP	16	2.95 ± 0.12
M-PEEP	32	2.67 ± 0.05^*^
H-PEEP	64	2.61 ± 0.09^*^

The data were expressed as the means ± S.E.M. (n = 5).^#^ *p* < 0.05 vs. Normal group. ^*^ *p* < 0.05, vs. CFA, group.

### 3.8 Effect of PEEP on the Levels of IL-6, PGE_2_ and TNF-α in Serum of Complete Freund’s Adjuvant Rats

**FIGURE 6:**
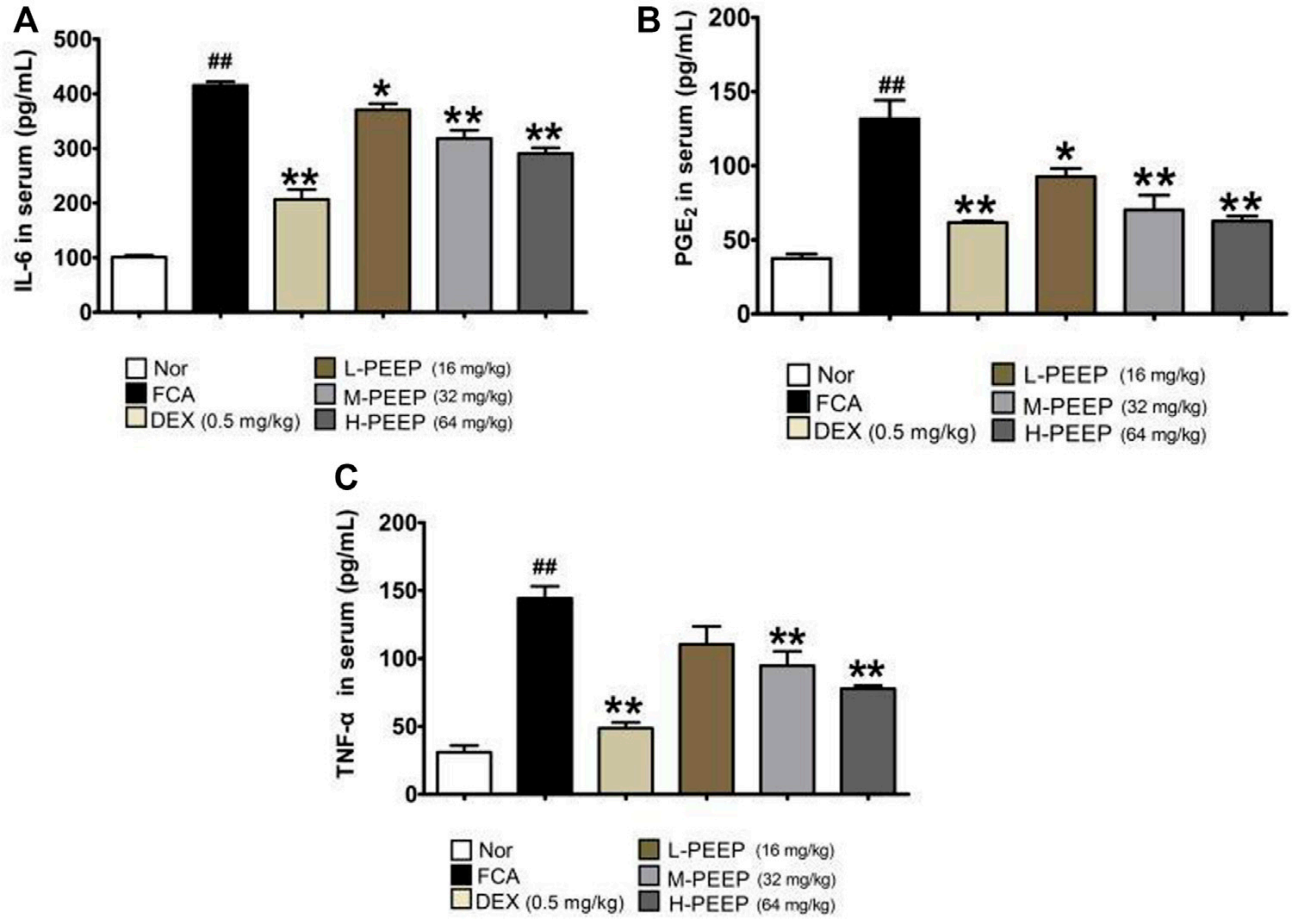
Effects of different doses of PEEP on the expression of inflammatory cytokines in CFA rat serum. **(A)** The concentrations of IL-6 in serum were determined by ELISA. **(B)** The concentrations of PGE2 in serum were determined by ELISA. **(C)** The concentrations of TNF-α in serum were determined by ELISA. The data were expressed as the means ± S.E.M. (*n* = 5). ^##^ *p* < 0.05 vs. Normal group; ^*^ *p* < 0.05, ^**^ *p* < 0.01 vs. CFA group.

The serum levels of IL-6, PGE_2_ and TNF-α in the model group increased significantly when compared with the blank group (*p* < 0.01). The expressions of IL-6 and PGE_2_ in the serum of rats in the L-PEEP (16 mg/kg) group, M-PEEP (32 mg/kg) group, and H-PEEP (64 mg/kg) group were significantly lower than the model group (*p <* 0.05). the expression of TNF-α in the serum of rats in the M-PEEP (32 mg/kg) group and H-PEEP (64 mg/kg) group was significantly reduced (*p* < 0.05), but the L-PEEP (16 mg/kg) group only had a decreasing trend, and the difference was not statistically significant (*p* > 0.05). Therefore, it can be considered that medium and high doses of PEEP can reduce the expression of IL-6, PGE_2_, and TNF-α, which can inhibit inflammation and control the development of the disease. The treatment effect of L-PEEP group was slightly worse than that of M-PEEP group and H-PEEP group (Figure 6).

### 3.9 X-Ray Observation Results of Rat Feet in Each Group

**FIGURE 7:**
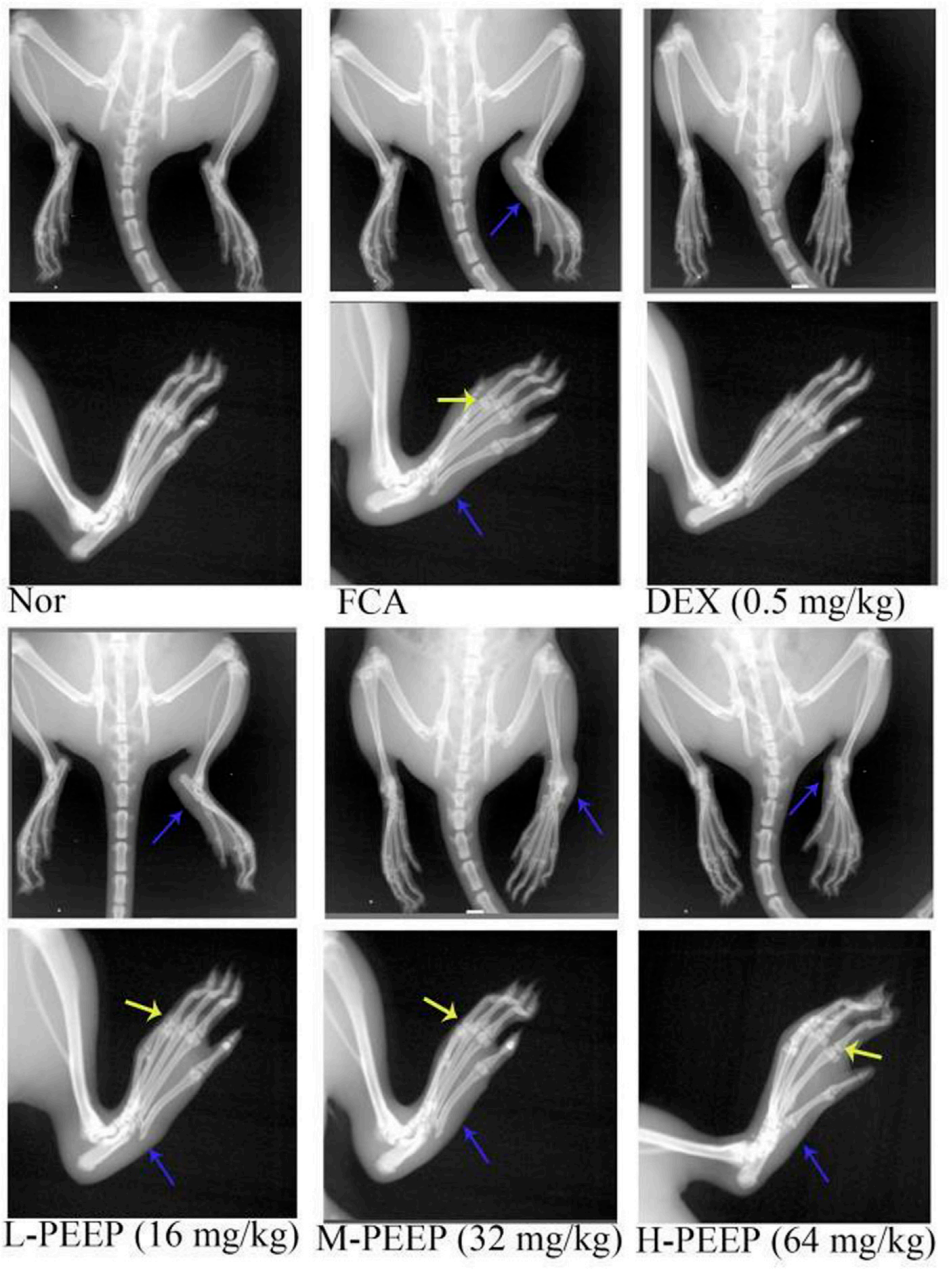
X-ray evaluation of joint injury in CFA rats. X-rays showed the effects of different doses of PEEP on hind paw and right paw of CFA rats. Blue arrow, swelling of soft tissue; yellow arrow, narrow joint space, blurred joint surface.

The paws of the rats in the model group were swollen, the joint space was narrow, and the articular surface was blurred. M-PEEP (32 mg/kg) group, H-PEEP (64 mg/kg) group and DEX group (0.5 mg/kg) reduced the swelling of soft tissue around the ankle joint and improved the bone surface erosion in rats and joint space narrowing after 10 days of administration by observing the X-ray of the right hind paw of the rats in each group. The left foot swelling of CFA rats in each group was not obvious, and there was no bone destruction (Figure 7).

### 3.10 Observation of PEEP on HE Staining of Ankle Joint of Complete Freund’s Adjuvant Rats

**FIGURE 8:**
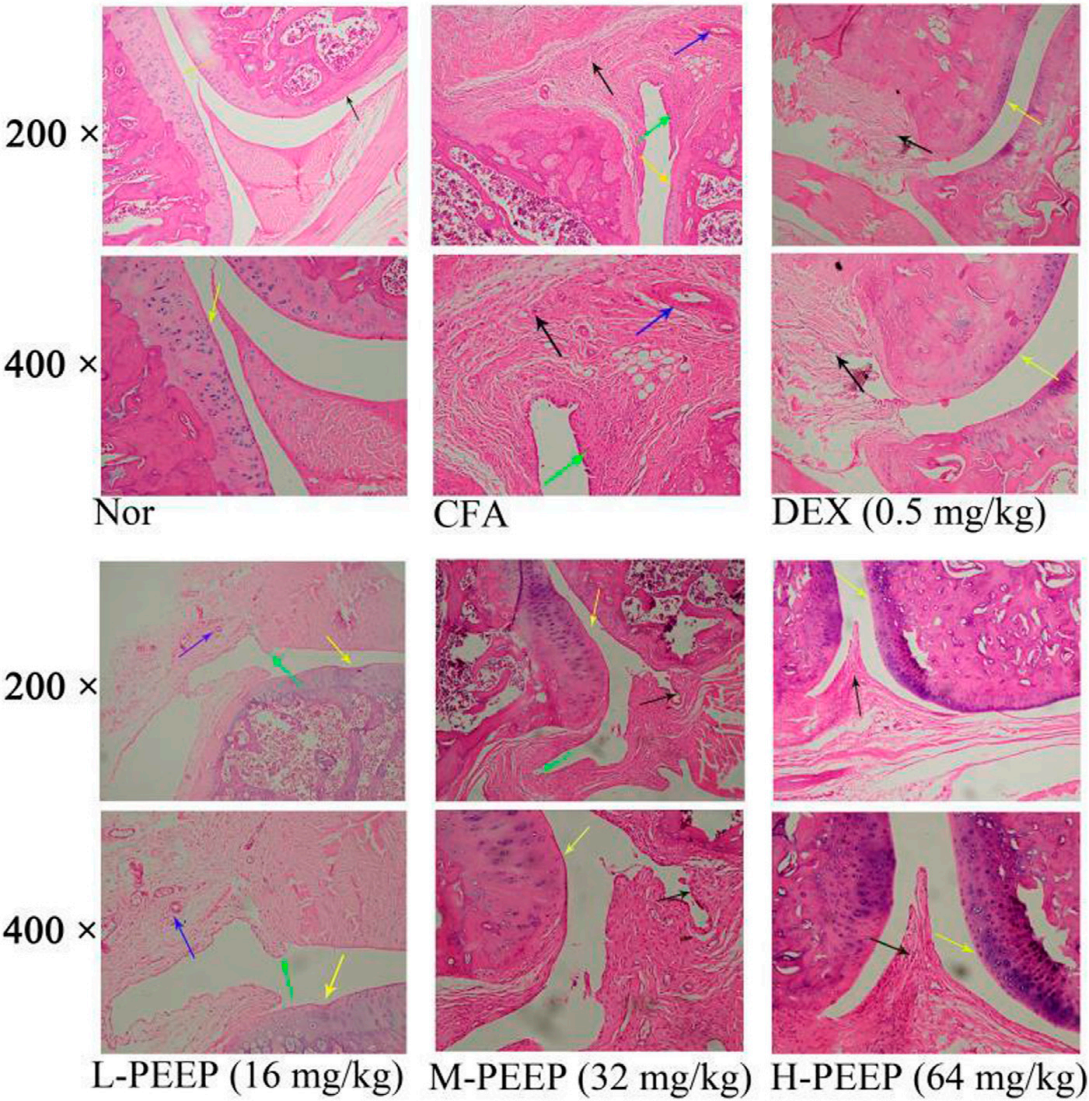
The pathologial slice pictures of the ankle joint of CFA rats treated with various doses of. PEEP. Red arrow, articular cartilage; black arrow, synovial hyperplasia; green arrow, inflammatory cell infiltration; blue arrow, pannus.

HE staining of the blank group showed complete and clear structure, no inflammatory cell infiltration and synovial hyperplasia, and no damage to cartilage and bone joints. Cartilage destruction, inflammatory cell infiltration, proliferation of a large number of synovial cells, and pannus formation occurred in the foot and ankle joints in the model group. HE staining in the DEX (0.5 mg/kg) group showed slight synovial hyperplasia and less inflammatory cell infiltration when compared with the model group. The degree of synovial hyperplasia or inflammatory cell infiltration in the M-PEEP (32 mg/kg) group and H-PEEP (64 mg/kg) group was lighter, and the cartilage erosion was lighter than the model group (Figure 8).

### 3.11 Effect of PEEP on p38/MAPK Signal Pathway in Complete Freund’s Adjuvant Rats

**FIGURE 9:**
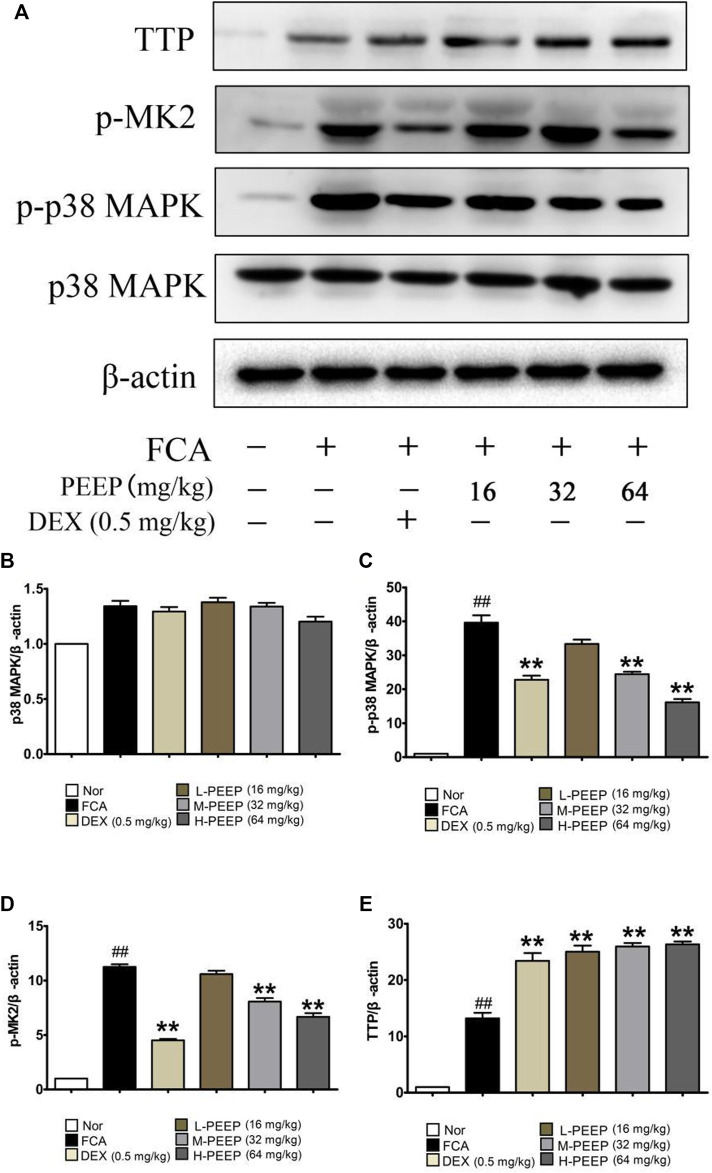
Effects of different doses of BGE on the p38/MAPK signaling pathway. **(A)** The expression of p-38, p-p38, p-MK2, and TTP obtained from ankle joints of several groups was detected by western blot analysis. **(B)** Quantitative analysis of gray value of p38 was performed in several groups with β-actin as loading control. **(C)** Quantitative analysis of gray value of p-p38 was performed in several groups with β-actin as loading control. **(D)** Quantitative analysis of gray value of p-MK2 was performed in several groups with β-actin as loading control. **(E)** Quantitative analysis of gray value of TTP was performed in several groups with β-actin as loading control. Data represent the mean ± S.E.M. of three independent experiments. *p* < 0.05, ^##^ *p* < 0.01 vs. Normal group; ^**^ *p* < 0.01 vs. CFA group.

The results are presented in Figure 9, the expression of p38 in the ankle joint has no significant difference (*p* > 0.05). The expression of p-p38 and p-MK2 in the M-PEEP (32 mg/kg) group and the H-PEEP (64 mg/kg) group was reduced when compared with the model group (*p* < 0.01), while the expression of p-p38 and p-MK2 in the L-PEEP (16 mg/kg) group has a decreasing trend, no significant difference (*p* > 0.05). The protein expression of TTP in the ankle joints increased when compared with the model group (*p* < 0.01). Overall, the results show that PEEP can inhibit the expression of p-p38 and p-MK2 proteins in the foot and ankle joints of CFA rats, and reduce the phosphorylation of TTP. There is a certain dose relationship, and the best effect is shown at high doses.

## 4 Discussion

The etiology and pathogenesis of RA have not been clearly elucidated. The number of macrophages is one of the main factors in RA because the severity of the disease and the degree of joint damage are significantly related to the number of macrophages (Kinne et al., 2007). When macrophages are stimulated by LPS, they will activate MAPK and other related signal pathways, and then secrete a large number of inflammatory factors, such as TNF-α, IL-6, IL-1β, and PGE_2_ (Xu et al., 2017; Yonezawa et al., 2018). RAW264.7 mouse macrophages are a type of macrophages. Because of their strong adhesion and ability to swallow antigens, they are generally used to build inflammation models and conduct research on inflammatory responses (Adams and Hamilton, 1984; Yang et al., 2014). LPS was used for stimulation to establish a macrophage inflammation model to study the effect of PEEP on inflammation and its molecular mechanism. According to the literature, the preliminary experimental results of the research group, 1 μg/ml LPS was used to stimulate RAW264.7 macrophages to construct as vitro inflammation model. The CCK-8 method was used to detect the effects of different concentrations of *P. aculeata* Mill on the proliferation of RAW264.7 macrophages. The results showed that, the concentrations of PEEP (0, 15, 30, and 60 μg/ml), EEEP (0, 5, 15, and 30 μg/ml), and BEEP (0, 10, 20, and 40 μg/ml) no significant effect on the survival rate of RAW264.7 macrophages when compared with the blank group. Therefore, PEEP (60 μg/ml), EEEP (30 μg/ml), and BEEP (40 μg/ml) are selected as the highest concentrations for subsequent experiments.

In the early stage of inflammation, inflammatory cytokines such as TNF-α and IL-6 released by activated macrophages play an extremely important role in initiating local inflammation (Ueda et al., 2015). TNF-α is a potent pro-inflammatory cytokine released by macrophages, and its secretion reflect the severity of inflammation in the body (Caldwell et al., 1999; Su et al., 2015), and the excessive release of TNF-α can induce the production of other cytokines (such as IL-6), to further amplify the damage and destruction of TNF-α to inflamed tissues. Therefore, inhibiting the expression of TNF-α has a good control effect on the inflammatory response (Jayaraman et al., 2008). IL-6 plays an important role in chronic inflammatory diseases, and promotes the proliferation and differentiation of cells in inflammation (Tanaka and Kishimoto, 2014; Takeuchi et al., 2021). It is associated with high levels of IL-6 in a variety of inflammation-related diseases. Therefore, IL-6 is used as an indicator of anti-inflammatory efficacy (Mesquida et al., 2014). NO is a highly reactive oxidative free radical in organisms, which is generated by endogenous L-arginine under the catalysis of iNOS (Raij, 2006). It has been reported in the literature that NO is directly involved in the inflammatory response and joint damage process of RA (Mccartney-Francis et al., 2001). High expression levels of NO can cause hyperdilatation and increased exudation of blood, accompanied by the release of inflammatory cytokines (TNF-α, IL-6). TNF-α induces the synthesis of iNOS and further increases the level of NO (Cohen et al., 2017). Therefore, the level of NO can also be used as one of the important indicators for observing the inflammatory response. The NO colorimetric method to detect the effect of different concentrations of *P. aculeata* Mill extracts on the LPS-induced NO expression in RAW264.7 macrophages. The results show that the extracts from *P. aculeata* Mill significantly reduce NO. PEEP has the most significant effect which is dose-dependent. It is suggested that PEEP more effectively reduce the production of NO, and play a better therapeutic effect on intracellular inflammation and oxidative stress. Next, the Q-PCR experiment was conducted to investigate the effects of each extraction site of *P. aculeata* Mill on the expression of inflammatory factors in RAW264.7 macrophages induced by LPS. The results showed that PEEP significantly inhibit TNF-α and IL-6. EEEP and BEEP have no significant effect on the levels of TNF-α and IL-6 in cells. Based on the experimental results, PEEP significantly reduce the levels of NO, TNF-α, and IL-6 in activated RAW264.7 macrophage expression level and inhibit cell inflammatory response.

p38/MAPK is an important member of the MAPK family. It triggers a chain reaction of intracellular protein kinases under the stimulation of inflammatory factors and external factors, thereby affecting biological effects such as cell transcription, protein synthesis and cell surface receptor expression (Munoz and Ammit, 2010). The p38/MAPK signaling pathway is one of the most important family members that control inflammation, and plays an important role in the physiological and pathological processes such as inflammation, cell stress, apoptosis, cell cycle and growth (Niranjan et al., 2012). Under LPS stimulation, macrophages and the p38/MAPK signaling pathway be activated, and cause the secretion of a large number of inflammatory factors (TNF-α, IL-6) (Zhu et al., 2016; Dong et al., 2018). The activation of p38/MAPK lead to the overexpression of p-MK2 which downstream substrate of the pathway, and activated MK2 phosphorylate zinc finger protein 36 (Tristetraprolin, TTP), thereby over-promoting and regulating cytokines such as TNF-α and IL-6 hyperplasia leads to the continuous expression of inflammatory factors and causes a series of inflammatory reactions. Therefore, the p38-MK2-TTP axis mediate pro-inflammatory signals, which also explains why the knockout mouse MK2 gene cannot be successfully prepared into experimental arthritis (Hegen et al., 2006). We explored the effects of different concentrations of PEEP on the expression of p-p38, p-MK2 and TTP in RAW264.7 macrophags stimulated by LPS in order to further explore the molecular mechanism of PEEP inhibiting inflammation. Western blot was used to detect the effect of PEEP on the p38/MAPK signaling pathway of RAW264.7 macrophages stimulated by LPS. The results showed that the expression of p38 in activated RAW264.7 macrophages was not significantly different The levels of p-p38 and p-MK2 in the cells induced by LPS increased significantly when compared with the blank group. The expressions of p-p38 and p-MK2 in PEEP (30 and 60 μg/ml) were lower than those in the model group. The expression of TTP in each PEEP administration group increased when compared with the model group. The results suggest that PEEP exerts anti-inflammatory effects by regulating the p38/MAPK signaling pathway. PEEP can reduce the expression of NO, TNF-α, and IL-6 in RAW264.7 macrophages induced by LPS. The mechanism may be through regulating the p38/MAPK signaling pathway, thereby inhibiting LPS-induced inflammatory response in RAW264.7 macrophages.

CFA-induced arthritis model is the basic method of the immune arthritis rat model. It is an inflammatory response mediated by immune complexes and its pathogenesis is mainly based on molecular simulation theory. Intradermal injection of CFA delays the body’s absorption and causes continuous stimulation of the body to produce a secondary autoimmune response (Shakeel et al., 2021). The primary lesion of adjuvant arthritis model rats is an acute stress response, it will appear red and swollen joints and foot soles after sensitization. The secondary lesions appeared about 10 days ago, which was an autoimmune reaction, generally manifested as the swelling of joints and plantar joints on the inflamed side and non-inflamed side after modeling. The rat adjuvant arthritis model established by CFA is a simple method, with high success rate and stable model after establishment. Its pathological process and laboratory indicators are similar to those of RA and it is an ideal model for screening and researching drugs for the treatment of RA (Wang et al., 2019). Therefore, we established an CFA-induced arthritis rat model in order to further verify the anti-RA effective part of *P. aculeata* Mill. The results of this experiment show that, rats in the model group were injected with CFA intracutaneously on the right hind foot plantar. The inflamed right paw of the rat began to swell within 5–9 h, and the swelling degree of the rat’s foot gradually became swollen. It swelled again 14 days after modeling, secondary lesions generally appear at about 11 days after inflammation, and the swelling of the contralateral foot gradually increases to a peak. *P. aculeata* Mill petroleum ether had a significant inhibitory effect on the swelling of the left and right paws of arthritis after 10 days of administration when compared with the model group.

RA mainly manifests as persistent joint and synovial inflammation, accompanied by bone and bone erosion, joint adhesion, pannus formation, and inflammatory cell infiltration (Turesson et al., 2003; Lee et al., 2012). HE staining and X-ray observation of rat foot and ankle joints showed that the synovial tissue of the model group was proliferated, inflammatory cells infiltrated, and pannus formed. X-rays showed that the soft tissues of the model group were swollen, the joint space was narrow, and the articular surface was blurred. PEEP significantly improve synovial hyperplasia, inflammatory cell infiltration and pannus formation in rat foot and ankle joint disease, reduce the swelling of soft tissue around the foot joint, and improve the bone surface erosion in rats and the degree of joint space stenosis after administration when compared with the model group.

The spleen is the body’s immune organ and plays a very important role in the body’s immune function (Xu et al., 2021). The results of this experiment showed that the spleen index of the model group increased. PEEP reduce the spleen index of CFA rats when compared with the model group. It shows that CFA rats have immune disorders and damaged immune organs. A certain dose of PEEP has a protective effect on the immune function of CFA rats.

In the process of inflammation in CFA rats, the release of inflammatory cytokines (TNF-α, IL-6, IL-8, etc.) played an important role in the occurrence and development of inflammation, the disintegration of articular cartilage matrix and bone destruction (Scott et al., 2010; Wang et al., 2017; Uttra et al., 2018). TNF-α is one of the earliest and most important inflammatory mediators in the process of inflammation. It can activate neutrophils and lymphocytes, increase the permeability of vascular endothelial cells, regulate the metabolic activity of other tissues and promote the synthesis and release of other cytokines (Moelants et al., 2013). PGE_2_ is an important inflammatory mediator as a second messenger. The inflammatory response is closely related to the part PGE_2_ expression level. Studies have shown that high concentrations of PGE_2_ will dilate capillaries, increase vascular permeability, tissue congestion and edema, aggravate inflammation, and even produce collagenase, erode cartilage, and destroy bone (Akaogi et al., 2006; Chen et al., 2012). Further studies have found that PGE_2_ induce the production of IL-6, and these cytokines usually interact with each other to form a network and jointly participate in the pathogenesis of RA (Matsuno et al., 2002; Axmann et al., 2009; Takeuchi et al., 2020). Therefore, it can help determine the improvement or deterioration of the inflammatory state in rats when studying the levels of TNF-α, IL-6, and PGE_2_. The results showed that the expression levels of inflammatory factors TNF-α, IL-6, and PGE_2_ in the serum of the model group increased significantly. PEEP reduce the levels of inflammatory factors TNF-α, IL-6, and PGE_2_ in the serum of rats when compared with the model group.

We explored the regulation of PEEP on the p38/MAPK signaling pathway by detecting the protein expressions of p38, p-p38, p-MK2, and TTP in the ankle joints. It preliminarily proves that PEEP regulate the p38/MAPK pathway by inhibiting the expression levels of p-p38 and p-MK2 and reduce the phosphorylation of TTP, and ultimately slow down the inflammatory response and the progression of RA disease.

Each group of PEEP can reduce the index of the spleen of the immune organ, significantly improve the synovial hyperplasia, inflammatory cell infiltration and pannus formation in the foot and ankle joint disease of rats, reduce the swelling of the soft tissue around the foot and ankle joint, and improve the bone of the foot and ankle joint in rats surface erosion and joint space stenosis alleviate various inflammations in rats with adjuvant arthritis. The mechanism of action is that PEEP downregulates the levels of TNF-α, IL-6, and PGE_2_ in rat serum by regulating the p38/MAPK pathway, thereby alleviating the RA inflammation and damage.

**FIGURE 10:**
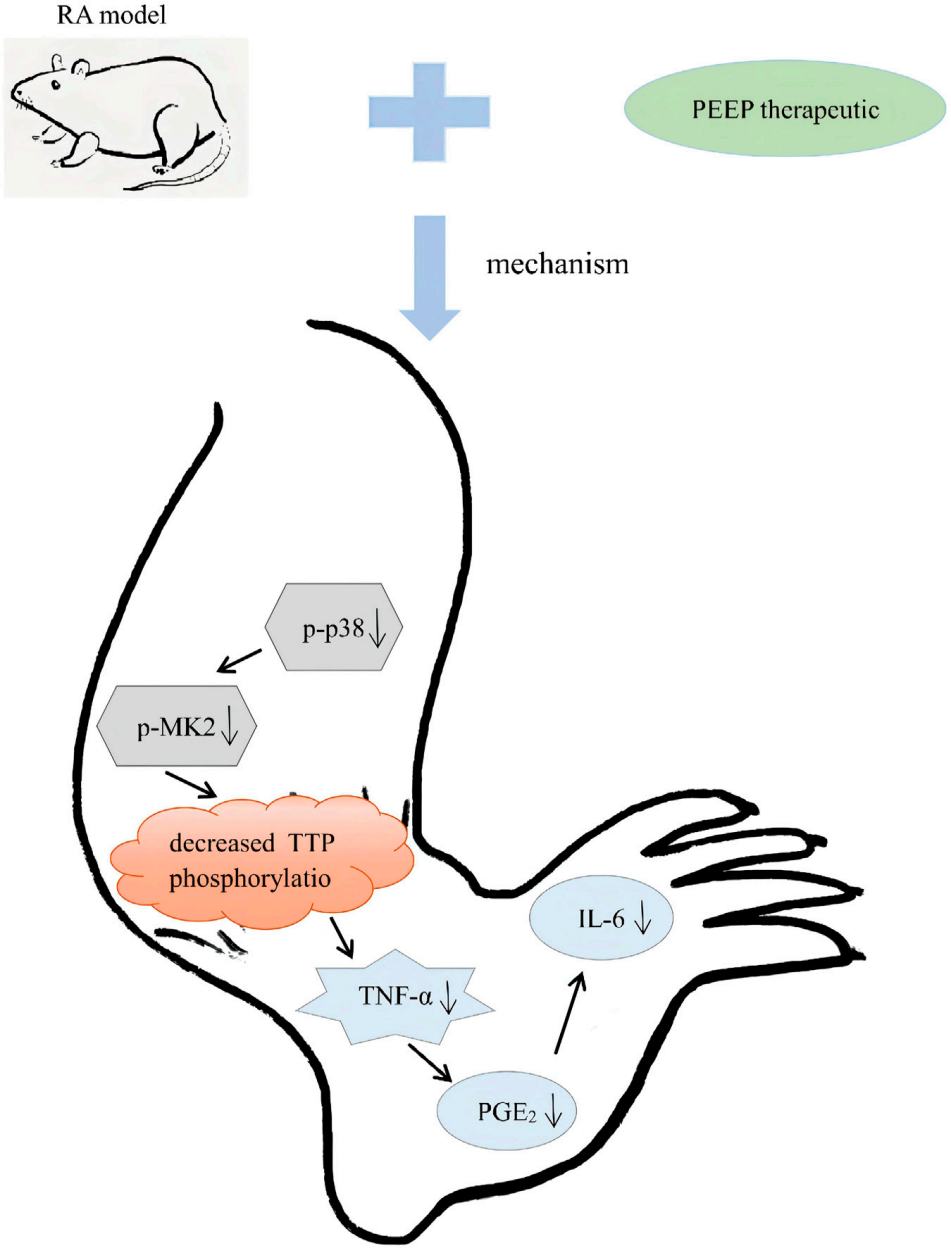
Schematic diagram of the mechanism of action of PEEP *in vivo*.

The pharmacodynamic evaluation *in vitro* and *in vivo* strongly demonstrated that the *P. aculeata* Mill petroleum ether site is the active site for anti-rheumatoid arthritis. *P. aculeata* Mill petroleum ether site regulate the p38/MAPK pathway by inhibiting the expression levels of p-p38 and p-MK2 and reduce the phosphorylation of TTP, and further inhibit the secretion of NO, TNF-α, IL-6, and PGE_2_ inflammatory cytokines, eventually slow down the inflammatory response and the progression of RA disease (Figure 10).

## Data Availability

The original contributions presented in the study are included in the article/Supplementary Materials, further inquiries can be directed to the corresponding authors.

## Abbreviations

BEEP, N-butanol Extract of Pereskia aculeate Miller; CCK-8, Cell Counting Kit-8; DEX, Dexamethasone; DMSO, DimethylsμLfoxide; EEEP, Ethyl Acetate Extract of Pereskia aculeate Miller; ELISA, Enzyme linked immunosorbent assay; CFA, Complete Freund’s adjuvant; IL-6, βinterleukin-6; LPS, Lipopolysaccharide; NO, Nitric oxide; PBS, Phosphate buffer asline; PEEP, Petroleum Ether Extract of Pereskia aculeate Miller; PGE2, Prostaglandin E2; p38MAPK, p38 mitogen-activated protein kinase; RA, Rheumatoid arithritis; TNF-α, Tumor necrosis factor α; TTP, Tristetraprolin.
